# Stent Fractures: New Insights into an Old Issue

**DOI:** 10.3390/jcm11020424

**Published:** 2022-01-14

**Authors:** Sa’ar Minha, David Pereg

**Affiliations:** 1 Interventional Cardiology, Shamir Medical Center, Be’er-Yaakov 6093000, Israel; 2 Sackler School of Medicine, Tel-Aviv University, Tel Aviv 6997807, Israel; davidpe@tauex.tau.ac.il; 3 Cardiology Department, Meir Medical Center, Kfar Saba 4428164, Israel

Percutaneous coronary intervention (PCI) is a safe and effective procedure performed worldwide providing both symptom relief and sustained improved outcomes for millions of patients. However, even in the present era of second- and third-generation drug-eluting stents, up to 20–25% of patients sustain a stent failure within 5 years. The etiology for stent failure is multifactorial and includes both patient- and device-related causes [1]. Stent failure is associated with a poor prognosis, recurrent symptoms, the need for re-interventions, a low quality of life, and increased health expenditure. While stent edge dissections, mal-apposition, and under-expansion are well-reported device-related etiologies for stent failure, stent fracture (SF) remains an elusive and less-discussed cause. The exact incidence of SF varies across different reports, ranging between 2 and 20%. Furthermore, the association between different SF patterns and outcomes is not sufficiently defined [2]. 

In the present Special Issue of the *Journal of Clinical Medicine*, Schochlow et al. shed new light on SF by utilizing retrospective optical coherent tomography (OCT) data. The study included two datasets: the first included 160 patients undergoing routine follow-up by OCT, while the second dataset included patients in whom OCT was performed due to stent failure. The prevalence of SF at 12 months was 30.8%, with lesion bifurcation and calcified lesions being independent predictors. In the dataset of 56 patients with stent failure, the prevalence of SF was 61.2%, with no difference in SF prevalence between patients presenting with restenosis and those presenting with stent thrombosis. In a multivariate logistic analysis, SF was associated with an OR of 12.5 (95%CI, 5.3–29.4; *p* < 0.001) for stent failure. When different patterns of SF were explored, a gap in the stent was associated with a 10 times higher risk for stent failure. Although this is a hypothesis-generating study, based on a relatively small-scale cohort, these findings are important and should promote a discussion regarding strategies for preventing SF and subsequent stent failure. As the population ages, the rate of complex PCIs including severely calcified and bifurcation lesions is expected to increase. As recommended by multiple consensus papers, when treating heavily calcified coronary lesions that appear resistant to conventional strategies, adequate lesion preparation using different calcium modification techniques such as scoring balloons, rotational atherectomy, or coronary lithotripsy should be employed prior to stent deployment. Following stent deployment, adequate post-dilatation and a proximal optimization technique should be employed, especially in bifurcation lesions. Schochlow et al. found an association of SF with coronary calcifications and asymmetrical stent expansion [3,4]. This association may suggest that, at least in part, these lesions were not adequately prepared prior to stenting. In such cases, stent deployment may be suboptimal and require post-stenting extremely high-pressure balloon dilatations, which can result in SF. The recently published 5-year follow-up of the SYNTAX II trial, which included patients with three-vessel disease requiring revascularization, demonstrated that the use of newer-generation stents, invasive coronary physiology assessment, and PCI optimization by intravascular imaging resulted in improved outcomes, including a lower rate of revascularization and all-cause mortality. It is thus reasonable to believe that applying these concepts, alongside the widespread use of designated tools for treating calcified lesions, will facilitate the improvement in PCI results and decrease the SF rate [5]. 

The diagnosis of SF remains a challenge even for the experienced interventional cardiologist. While fluoroscopy remains the most common mode for SF diagnosis [2], novel X-ray enhancement techniques (e.g., stent-boost; Philips Medical Systems, Eindhoven, the Netherlands) have allowed an improved appreciation of SF [6]. However, intravascular imaging by OCT or intravascular ultrasound is still the most reliable mode for the diagnosis and severity assessment of SF. The relatively high SF prevalence observed in the present study, both in the short term and, more importantly, at the 12-month routine follow-up, highlights the high spatial resolution and sensitivity of OCT. As with stent edge dissections, the use of OCT allows the identification of even minor anatomical and technical issues before and after PCI. However, the clinical relevance of these findings is not well established. 

Finally, the treatment of SF is another issue that should be assessed in future studies. It seems reasonable that SF with significant deformations or transections of the stent should be treated with further angioplasty. Currently, data regarding which SFs should be treated and how are limited, but some reassurance can be drawn from a relatively large report that demonstrated similar target lesion revascularization rates in patients treated by stents or balloons for SF-related and non-SF-related in-stent restenosis. Moreover, [7] in patients at low risk of bleeding, with a diagnosis of stent thrombosis due to SF, a more potent P2Y12 inhibitor (prasugrel or ticagrelor rather than clopidogrel) should be preferred, and an extension of dual anti-platelet therapy beyond the first year should be considered [8].

In conclusion, SF remains an important and relevant etiology for stent failure and the need for re-intervention. While it seems reasonable that PCI optimization techniques and technologies should lead to a decrease in SF incidence, this should be investigated in further studies.
